# Neural substrates of continuous and discrete inhibitory control

**DOI:** 10.1038/s41398-022-02295-0

**Published:** 2023-01-24

**Authors:** Jonathon R. Howlett, Heekyeong Park, Martin P. Paulus

**Affiliations:** 1 VA San Diego Healthcare System, San Diego, CA USA; 2 Department of Psychiatry, University of California San Diego, La Jolla, CA USA; 3 Laureate Institute for Brain Research, Tulsa, OK USA; 4 University of North Texas at Dallas, Dallas, TX USA

**Keywords:** Biomarkers, Neuroscience, Human behaviour

## Abstract

Inhibitory control dysfunctions play an important role in psychiatric disorders but the precise nature of these dysfunctions is still not well understood. Advances in computational modeling of real-time motor control using a proportion–integral–derivative (PID) control framework have parsed continuous motor inhibition into a preemptive drive component (signified by the *K*_p_ parameter) and a reactive damping component (signified by the *K*_d_ parameter). This investigation examined the relationship between inhibitory control processing during a stop signal task and continuous motor control during a simulated one-dimensional driving task in a transdiagnostic sample of participants. A transdiagnostic psychiatric sample of 492 individuals completed a stop signal task during functional magnetic resonance imaging and a simple behavioral motor control task, which was modeled using the PID framework. We examined associations between the *K*_p_ and *K*_d_ parameters and behavioral indices as well as neural activation on the stop signal task. Individuals with higher damping, controlling for a drive, on the driving task exhibited relatively less strategic adjustment after a stop trial (indexed by the difference in go trial reaction time and by stop trial accuracy) on the stop signal task. Individuals with higher damping, controlling for a drive, additionally exhibited increased activity in the frontal and parietal regions as well as the insula and caudate during response inhibition on the stop signal task. The results suggest that computational indices of motor control performance may serve as behavioral markers of the functioning of neural systems involved in inhibitory control.

## Introduction

Inhibitory control dysfunctions are implicated in a range of psychiatric problems including anxiety [1], mood disorders [2], substance use disorders [3], and attention deficit hyperactivity disorder (ADHD) [4]. The neural substrates underlying inhibitory control comprise prefrontal regions, dorsal anterior cingulate cortex (dACC), inferior frontal gyrus (IFG), and presupplementary motor area (pre-SMA) and subcortical regions including the subthalamic nucleus (STN) [5]. Despite progress, individual markers of inhibitory control function have had limited utility in clinical contexts, likely in part because of the poor reliability of measures of inhibitory control across a range of behavioral paradigms [6].

Limited reliability of measurement has also likely hampered advances in theoretical understanding of the subcomponents of inhibition and inhibitory control and their interrelationships, contributing to inconsistent findings of the relationship between inhibition and clinical disorders. For example, anxiety has historically been associated with both an *excess* of behavioral inhibition [7] as well as a *deficit* in inhibitory control [1]. While excessive response inhibition has been proposed as a promising marker of clinical anxiety, the overall literature remains inconsistent [8]. The inconsistent relationship between inhibition and anxiety may be related to the observation that individuals dynamically make strategic adjustments to inhibitory demands based on their performance and context [9, 10]. For example, when the expectation of the need to inhibit is high, individuals will preemptively slow responses even before an explicit signal that response inhibition is needed [11]. This preemptive process has been termed proactive inhibitory control, in contrast to reactive inhibitory control occurring after a stop signal [12, 13]. Anxious individuals may exhibit exaggerated proactive inhibitory control coupled with decreased ability to quickly inhibit in response to specific perceptual signals. Beyond anxiety, reliable measures of subcomponents of inhibitory processing may be useful as markers of core processing dysfunctions across multiple psychiatric diagnoses, which could lead to more mechanistically informed approaches toward assessment and treatment.

In parallel to the literature on the inhibition of discrete motor responses, recent research has examined motor control in continuous-time settings. Far from being a simple, mechanical process, human motor control represents a dynamic, computationally rich real-time decision process that incorporates high-level reward and threat information in a nuanced, nearly optimal manner [14, 15]. We have applied a proportion–integral–derivative (PID) control modeling approach to measure deficits in anxious individuals on a simple, simulated driving task [16]. This approach enabled highly reliable estimation of two individual parameters: *K*_p_, a proportion or drive parameter that increases acceleration in proportion to the current distance from a goal state, and *K*_d_, a derivative or damping parameter that reduces acceleration in proportion to velocity toward the goal, preventing overshoot. *K*_p_ reflects the processing of the current error, while *K*_d_ reflects the processing of the anticipated error. High *K*_p_ coupled with high *K*_d_ enables a rapid goal approach with minimal overshoot. Because velocity changes more quickly than position, derivative control requires rapid motor inhibition in response to incoming perceptual information to reduce anticipated error, similar to reactive inhibitory control on a stop signal task. Individuals with deficient derivative control may compensate by reducing *K*_p_, preemptively slowing to prevent overshoot, as in proactive inhibitory control. We found that low *K*_p_ and low *K*_d_ were associated with fear and with a low volume of the dACC, a region implicated in affective influence on motor control [17], inhibitory control [5], and fear expression [18]. These findings suggest that anxiety involves general proactive motor inhibition coupled with a deficit in rapid inhibition in response to perceptual information. Critically, split-half comparisons revealed that PID model parameters were estimated with extremely high reliability, unlike for traditional inhibitory control paradigms (*r* = 0.98 for *K*_p_ and *r* = 0.95 for *K*_d_).

The similarities between traditional inhibitory control paradigms and the sensorimotor driving paradigm, and the common role of the dACC, suggest that a sensorimotor paradigm may usefully probe neural deficits in inhibitory control, but with much more reliable indices potentially more useful in individualized clinical assessments. To test the relationship between real-time motor control and discrete motor inhibition, we compared behavioral and neural indices from a stop signal paradigm performed in an fMRI scanner with PID model parameters from a sensorimotor paradigm in a sample that included healthy volunteers and a transdiagnostic group of individuals with mood and anxiety complaints, substance use disorders, or eating disorders. We hypothesized that low *K*_p_ and low *K*_d_ would be associated with impaired recruitment of inhibitory control in response to a stop signal as well as with increased preemptive slowing when the expectation of the need to stop was high.

## Materials and methods

### Participants

The experiment was part of the Tulsa-1000 (T-1000; ClinicalTrials.gov NCT02450240) study, a naturalistic longitudinal study of individuals with mood, anxiety, substance use, and/or eating disorders, along with healthy volunteers (with sample size being based on power analyses to detect effects based on conservative assumptions) [19]. The target population was comprised of a Mood/Anxiety group including individuals with Patient Health Questionnaire (PHQ-9) [20] ≥10 and/or Overall Anxiety Severity and Impairment Scale (OASIS) [21] ≥8, a Substance Use group including individuals with Drug Abuse Screening Test (DAST-10) [22] score ≥3, an Eating Disorders group with Eating Disorder Screen (SCOFF) [23] score ≥2, and a Healthy Control group with individuals who did not screen positive for any of the above inclusion criteria. 492 individuals (age: 34.20 ± 10.55 years; gender: 176 male and 316 female) participated in the experiment, of whom 255 were in the Mood/Anxiety group, 153 were in the Substance Use group, 27 were in the Eating Disorders group, and 57 were in the Healthy Control group (see Table 1). 3 subjects were excluded for incomplete behavioral data on the stop signal task and 2 were excluded for incomplete sensorimotor task data. As part of the T-1000 study, participants completed self-report measures including the behavioral inhibition system (BIS) scale [24] and neuropsychological testing which included color-word inhibition. All study procedures were approved by the Western Institutional Review Board, and all participants provided written informed consent prior to participation.

**Table 1:** Demographic and clinical characteristics of the study sample.

	Healthy volunteers (*N* = 57)		Clinical population (*N* = 435)		*p*-value
Age, *M* (SD)	32.2	(11.2)	34.6	(10.5)	0.11
Male, *N* (%)	28	(49%)	148	(34%)	0.04
Race/Ethnicity, *N* (%)					0.09
White	41	(72%)	285	(69%)	
American Indian or Alaska Native	5	(9%)	83	(19%)	
Black or African American	2	(4%)	28	(7%)	
Hispanic	4	(7%)	16	(4%)	
Asian or Pacific Islander	2	(4%)	3	(1%)	
“Other”, unspecified	3	(5%)	18	(4%)	
Years Education, *M* (SD)	6.7	(1.6)	5.8	(1.9)	0.001
Income, *M* (SD)	$57,047	($47,508)	$45,422	($72,524)	0.24
PHQ-9, *M* (SD)	0.82	(1.20)	10.34	(6.17)	<0.001
OASIS, *M* (SD)	1.12	(1.39)	8.32	(4.40)	<0.001
DAST, *M* (SD)	0.12	(0.38)	3.18	(3.72)	<0.001
SCOFF, *M* (SD)	0.09	(0.29)	1.00	(1.26)	<0.001

*PHQ-9* Patient Health Questionnaire, *OASIS* Overall Anxiety Severity and Impairment Scale, *DAST* Drug Abuse Screening Test, *SCOFF* eating disorders screening questionnaire.

### Sensorimotor task

Participants performed a simulated one-dimensional driving task (16). Behavioral and computational modeling results from this task have previously been reported for the Mood/Anxiety and Healthy Control groups from the T1000 study [16]. Participants completed 30 trials of the task, with each trial having a fixed duration of 10 s. Participants were instructed to drive a virtual car, controlled using a gaming joystick, as quickly as possible and stop as close as possible to a stop sign without crossing the stop-line. The car was controlled according to a linear dynamical system, in which car velocity was proportional to joystick displacement at each time point. Throughout each trial, joystick displacement was recorded with a sampling window of 1/60 s.

### Stop signal task

Participants were instructed to make a response (go trials) or to cancel the response (stop trials) following a task cue in the stop signal task (SST) (22). The SST consisted of 6 blocks of 48 trials, each of which contained 36 go trials (75%) and 12 stop trials (25%) in pseudo-random order, for a total of 288 trials. Each block was separated by 12 s, and each trial lasted 1300 ms with a 200 ms intertrial interval. At the beginning of each trial, a black cue (‘X’ or ‘O’) appeared on a white background. On ‘go’ trials, participants were instructed to press, as quickly as possible, the left button for an ‘X’ cue and the right button for an ‘O’ cue. On ‘stop’ trials, in which a tone was presented and the task cue color changed to red, participants were instructed not to press either button. Prior to scanning, participants completed a practice run of the task to determine their mean response times (RTs) from the onset of the cue. The mean RT was used to determine the delivery time of the tone for the stop signal. Stop signals were delivered 500, 400, 300, 200, 100, or 0 ms less than the mean RT. Due to the difficulty of making a response in a short time, the stop trials with 0, 100, and 200 ms delays were counted as the short stop signal delay (SSSD) or difficult condition whereas the stop trials with 300, 400, and 500 ms delays were counted as the long stop signal delay (LSSD) or easy condition.

### PD control model

For each participant, we estimated the parameters of a PD control model. We fit a PD rather than a full PID model due to our previous findings that this simpler model results in a superior fit, because the task design does not include a constant disturbance, making the integral control component unnecessary [16]. PD model parameters were estimated using R [25]. At each time point within a trial, acceleration was modeled as a linear combination of the current error (goal position minus current car position) and derivative of the error, with coefficients *K*_p_ and *K*_d_, respectively. *K*_p_ and *K*_d_ were estimated using linear regression for each trial, with each time point as a data point with acceleration as the dependent variable and error and derivative as predictors. See the supplement for details of model validation procedures (simulations and parameter recovery and split-half reliability).

### Behavioral analysis

Statistical analyses were performed in R [25]. Data were inspected to ensure assumptions of statistical tests were met. Given the high correlation between *K*_p_ and *K*_d_, we first computed the residualized *K*_d_ after controlling for *K*_p_ for each participant. We previously found that residualized *K*_d_ displayed high split-half reliability (*r* = 0.89) [16].

To assess the relationship between PD model parameters and strategic adjustments on go trials, we first computed the difference in response times on go trials occurring immediately after a stop trial and those occurring immediately after a go trial for each participant. This difference in reaction times was then used as a dependent variable in linear regressions with predictors including either *K*_p_ or residualized *K*_d_ along with age, gender, and years of education.

To assess the relationship between PD model parameters and strategic adjustments on stop trials, we computed the difference in the proportion of successful stops (stop accuracy) on stop trials occurring immediately after another stop trial and those occurring immediately after a go trial for each participant. This difference was used as a dependent variable in linear regressions with predictors including either *K*_p_ or residualized *K*_d_ along with age, gender, and years of education.

To examine the relationship between PD model parameters and other measures of inhibition, we performed four linear regressions with either the BIS scale or color-word inhibition score from neuropsychological testing as dependent variables and predictors including either *K*_p_ or residualized *K*_d_ along with age, gender, and years of education.

### fMRI data preprocessing and analysis

Imaging data were collected with two identical GE MR750 3 T scanners equipped with 8 RF channel phased array coils, at the same site. T1-weighted anatomical images were acquired in the 3D high-resolution, MP-RAGE pulse sequence (TR/TE = 5/2.012 ms, 0.9 mm-thick 186 axial slices, FOV 240 × 192 mm). Two hundred fifty-six volumes (2.9 mm thick, 39 slices, 1.875 × 1.875 mm voxels) of T2*-weighted echo-planar images (TR/TE = 2000/27 ms, axial plane, flip angle 78°, FOV 240 × 240 mm) were collected during the stop signal task. The Analysis of Functional NeuroImages software suite (AFNI, http://afni.nimh.nih.gov) was used for preprocessing and statistical analyses of imaging data.

The first three EPI volumes were discarded for signal stabilization. Then, images were subjected to despiking, slice-time correction (first slice), co-registration (T1-weighted image), motion-correction (ENORM > 0.3), normalization (MNI space, 2 mm^3^ voxels), and smoothing (4 mm^3^ FWHM) for preprocessing.

For analysis, a mixed-effects model was used to examine the association between *K*_d_, the damping parameter, and neural activity for response control on the stop signal task. Three events were constructed on a participant to model response control: ‘Go’ (go trials), ‘SSSD’ (short stop signal delay or difficult trials), and LSSD (long stop signal delay or easy trials). To avoid the influence of differential error rates by trial type, only correct responses were taken for analysis. Incorrect responses were modeled as no-interest events with motion parameters. The hemodynamic response for a trial was convolved with a gamma function from the onset of the task cue, in the whole brain.

The main contrast was constructed by comparing the “Stop” trials (SSSD + LSSD) versus the “Go” trials to model the neural response for control on the stop signal task. Then, the association between the model parameter, *K*_d_, and neural response for control (Stop > Go) was examined in a multivariate model with covariates of the individual’s diagnostic category (see Table 1), age, and sex, after controlling *K*_p_. The FDR of *p* < 0.05 was set for multiple testing corrections with a voxel-wise threshold of *p* < 0.001. Significant cluster effects (*k* > 60) were subjected to follow-up tests to probe the association between *K*_d_ and the difficulty level of control. Beta coefficients extracted from clusters were standardized (*M* = 0, SD = 1).

To determine whether *K*_d_ was associated with inhibitory control in depression and anxiety as well as in the broader transdiagnostic group, we also performed a separate analysis for individuals with major depression disorder, anxiety, and comorbid depression and anxiety.

To examine the specificity of the association between neural markers of inhibitory control and *K*_d_ vs. other measures of inhibition, whole-brain analyses were also performed as above with BIS score and with color-word inhibition substituted for *K*_d_.

## Results

### Behavioral results

*K*_p_ was not associated with the difference in go trial response times occurring after a stop trial or after a go trial (controlling for age, gender, and education), i.e., *K*_p_ was not associated with the behavioral index of strategic adjustments on go trials (*β* = −0.06, *p* = 0.19). Residualized *K*_d_ was negatively associated with the difference in go trial response times occurring after a stop trial or after a go trial. That is, those individuals with greater residualized *K*_d_ showed relatively less strategic adjustment in terms of response times during go trials (*β* = −0.14, *p* < 0.01).

*K*_p_ was not associated with the difference in stop trial accuracy occurring after a stop trial or after a go trial, showing no association with the behavioral index of strategic adjustments on stop trials (*β* = −0.05, *p* = 0.25). Residualized *K*_d_ was negatively associated with the difference in stop-trial accuracy occurring after a stop trial or after a go trial (controlling for age, gender, and education). Thus, similar to go trials, those individuals with greater residualized *K*_d_ showed relatively less strategic adjustment in terms of accuracy during stop trials (*β* = −0.12, *p* < 0.01).

*K*_p_ was not associated with the BIS scale (*β* = −0.06, *p* = 0.19), while residualized *K*_d_ was negatively associated with the BIS scale (*β* = −0.11, *p* < 0.01). *K*_p_ was positively associated with the color-word inhibition score (*β* = 0.16, *p* < 0.01), while residualized *K*_d_ was not associated with the color-word inhibition score (*β* = 0.07, *p* = 0.13).

### fMRI results

**Fig. 1:**
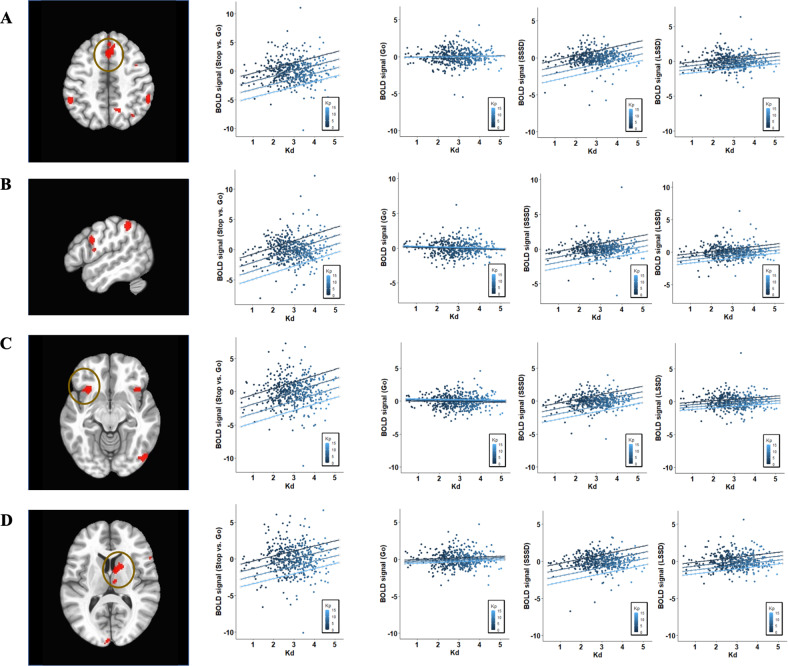
Brain regions showing associations between *K*_d_ and response control (Stop vs. Go). **A** R inferior frontal gyrus, **B** L Inferior parietal sulcus, **C** L aInsula, and **D** R Caudate. SSSD short stop signal delay or difficult control, LSSD long stop signal delay or easy control.

Imaging analyses were performed for 450 subjects with complete fMRI data. First, we examined the association between *K*_d_ and overall response control (Stop > Go) in the brain. Table 2 displays the brain regions showing significant associations between *K*_d_ and neural activity for control responses upon the stop signal. There was a positive association between *K*_d_ and several frontal cluster activities during response control. That is, those individuals with greater *K*_d_ showed greater activation in frontal regions for canceling responses. For instance, activation in the inferior frontal gyrus extending to the ventromedial part of the left frontal cortex exhibited a positive relationship between *K*_d_ and successful stopping responses (*β*_1_ = 0.94, 95% CI [0.43,1.45], *β*_2_ = 0.96, 95% CI [0.43,1.49]). In the right brain, both the inferior frontal gyrus and middle/medial parts of the frontal gyrus revealed the same pattern of a positive relationship between *K*_d_ and response control (*β*_1_ = 0.94, 95% CI [0.42,1.46], *β*_2_ = 0.84, 95% CI [0.36,1.31], *β*_3_ = 0.98, 95% CI [0.48,1.48]). At the subcortical level, the bilateral anterior insula and right caudate also showed positive correlations between the damping parameter and neural activity for stopping the default Go response (*β*_1_ = 1.06, 95% CI [0.52,1.60], *β*_2_ = 0.85, 95% CI [0.33,1.37], *β*_3_ = 0.78, 95% CI [0.25,1.30]). Further, bilateral parietal regions extended from superior to inferior parts revealed the direct relationship between neural control for inhibiting responses and the damping parameter, *K*_d_ (*β*_1_ = 1.13, 95% CI [0.59,1.66], *β*_2_ = 0.89, 95% CI [0.36,1.42]). Figure 1 displays the relationship between *K*_d_ and response control (Stop > Go), with individual events (Go, SSSD, and LSSD).

**Table 2:** Brain regions showing the relationship between *K*_d_ and control activity during Stop-Signal Task.

	Peaks (*x*,*y*,*z*)			# of voxels	Region	*t*-statistic
Stop > Go	3	23	47	237	R ventromedial frontal cortex	5.26
	−53	−45	49	234	L inferior parietal gyrus	5.29
	9	5	11	147	R caudate	4.56
	−55	5	31	145	L inferior frontal gyrus	4.98
	59	−39	43	139	R inferior parietal gyrus	5.07
	−31	21	−11	112	L anterior insula	5.21
	39	3	31	105	R inferior frontal gyrus	4.76
	31	7	59	100	R middle frontal gyrus	5.17
	57	11	17	88	R medial frontal gyrus	5.11
	39	21	−11	66	R anterior insula	4.72

To query the relationship between *K*_d_ and control difficulty, we compared LSSD versus Go trials (easy control) and SSSD versus Go trials (difficult control) and examined the relationship between *K*_d_ and easy or difficult level of controlling responses in the above Stop > Go clusters (Supplemental Fig. 1). Difficult control (SSSD > Go) was positively associated with *K*_d_ in all clusters showing the Stop > Go differences in a very similar way (Table 3). However, we did not find the relationship between *K*_d_ and easy control (LSSD > Go) in the Stop > Go clusters even at the uncorrected *p* < 0.001 threshold. Similarly, at the whole brain level, no suprathreshold cluster showed a relation between easy control and *K*_d_, while several clusters revealed an association between difficult control and *K*_d_ (Supplemental Table 1). Most of the clusters from the difficult control contrast at the whole brain level overlapped with the regions found for the association of *K*_d_ in overall response control (Stop vs. Go). The comparison between SSSD versus LSSD did not yield any significant cluster.

**Table 3:** The association of *K*_d_ and difficult control (SSSD > Go) in the clusters for Stop control (Stop > Go).

Region	Beta (CI)	*t*-statistic
R ventromedial frontal cortex	0.66 (0.35,0.97)	5.30
L inferior parietal gyrus	0.68 (0.36,0.99)	4.82
R caudate	0.53 (0.20,0.86)	4.38
L inferior frontal gyrus	0.59 (0.28,0.91)	4.64
R inferior parietal gyrus	0.57 (0.26,0.88)	4.88
L anterior insula	0.75 (0.40,1.09)	5.21
R inferior frontal gyrus	0.55 (0.24,0.85)	4.29
R middle frontal gyrus	0.47 (0.18,0.76)	4.29
R medial frontal gyrus	0.61 (0.30,0.92)	4.61
R anterior insula	0.67 (0.34,1.01)	5.03

To examine the association between *K*_d_ and inhibitory processing in depression and anxiety, we performed an analysis with individuals with major depression disorder, anxiety, and comorbid depression and anxiety (*n* = 236). Neural activity for inhibitory control (Stop > Go) associated with *K*_d_ was shown in fronto-parietal regions and bilateral insula (Table 4). All of these clusters showed positive relationships between *K*_d_ and neural activity in the clusters (Supplemental Fig. 2).

**Table 4:** Clusters showing the relationship between *K*_d_ and overall control (Stop vs. Go) in individuals with mood disorders.

Peaks (*x*,*y*,*z*)			Voxels	Region	*β* (95% CI)	*t*-statistic
41	−63	−23	375	R inferior/temporal occipital cortex	2.29 (1.42, 3.15)	5.23
1	23	47	368	L/R superior medial cortex	1.68 (0.91, 2.46)	4.30
−29	21	−11	205	L anterior insula	2.39 (1.57, 3.21)	5.76
−19	21	53	165	L middle/superior frontal cortex	2.13 (1.31, 2.95)	5.11
−51	−45	51	128	L inferior parietal lobe	1.94 (1.13, 275)	4.71
25	23	−3	123	R anterior insula	1.77 (0.92, 2.62)	4.09
−9	−83	−11	112	L lingual gyrus	1.20 (0.50, 1.90)	3.39
−27	−51	41	95	L inferior parietal lobe	1.47 (0.69, 2.24)	3.74
−5	−65	65	89	R precuneus	1.36 (1.42, 3.15)	3.79
−51	37	−9	86	L pars orbitalis	1.96 (1.17, 2.75)	4.87
−3	−17	−17	71	midbrain	2.26 (1.37, 3.16)	5.00
41	55	−1	63	R middle fronto-orbital gyrus	1.64 (0.87, 2.41)	4.20

Whole-brain analyses performed as above but with BIS score and with color-word naming inhibition score substituted for *K*_d_ yielded no significant clusters.

## Discussion

This investigation examined the relationship between inhibitory control processing during a stop signal task and continuous motor control during a simulated one-dimensional driving task in a transdiagnostic sample of participants. Behaviorally, individuals with higher levels of *K*_d_ controlling for *K*_p_ (i.e., higher levels of damping controlling for drive) on a continuous motor task were less likely to slow down preemptively after a stop trial on a stop signal task. Similarly, these individuals were less influenced by a previous stop trial in their performance on a current stop trial. These findings suggest that individuals with higher levels of damping on a continuous motor task rely less on proactive inhibitory control on a discrete motor inhibition task. *K*_d_ controlling for *K*_p_ was negatively associated with BIS score (a measure of behavioral inhibition rather than response inhibition), while *K*_p_ was positively associated with color-word inhibition score. On imaging, individuals with higher *K*_d_ controlling for *K*_p_ showed increased activity in frontal and parietal cortical regions, known for the fronto-parietal network for executive control and attention, as well as subcortical activity in insula and caudate for response control and selective inhibition [26, 27] during response inhibition. Similar findings emerged when the analysis was restricted to individuals with clinical depression or anxiety, while parallel analyses found no association between BIS or color-word inhibition and neural activation. Taken together, these associations between a computational parameter quantifying damping during continuous motor control and trial-based response time as well as brain activation during inhibitory control support the idea that a simple virtual driving task together with a well-developed computational framework is suitable to probe proactive or reactive inhibitory control and adds important information beyond traditional self-report and neuropsychological measures such as BIS and color-word inhibition.

The fronto-parietal network is well known for the cognitive control of both internal plans and the external environment with proactive and reactive processing (see ref. [28] for review) in addition to its well-established role in attention and executive control for goal-driven behavior [29, 30]. Top-down modulation of other brain areas for efficient processing of information and adjusting behavior flexibly is the critical contribution of the fronto-parietal control network. The present finding of the positive association between the damping parameter and the regions in the fronto-parietal network demonstrates that the damping parameter may represent cognitive control beyond inhibitory control of motor responses. In fact, it has been suggested that the fronto-parietal network plays a critical role in cognitive control as a flexible hub for both resting state and task states and that dysfunction of this network is implicated in many different psychopathological diseases [29].

The involvement of the insula and caudate in successful response control and the association with the damping parameter complements the possibility that the damping parameter serves as a computational marker for cognitive control. Activity in the insula and caudate have been observed in various tasks requiring cognitive control such as monitoring/processing conflicts, associating information, and selecting responses [31, 32]. Insula and caudate have also been functionally implicated in successful stop trials in SST as well as general task performance, with the involvement of the anterior insula in stopping efficiency [26]. We previously found that damping performance was positively related to caudal anterior cingulate cortex volumes [16]. Given that inhibitory control at least is hardly the function of a single brain region or structure but is more likely to be dependent on the interactions of multiple brain regions/structures, the association of *K*_d_ with neural activity in the fronto-parietal network as well as the anterior insula and caudate suggests that damping may include complex processing of cognitive control for contextually inappropriate behaviors. While future studies are warranted to test this interpretation, the present results shed further light on the neural underpinnings of damping by demonstrating an association between the damping parameter and neural activity in control-related regions during response inhibition.

PD control parameters may provide robust behavioral measures of facets of inhibition that have been obscured due to the limited reliability of measurements. For example, behavioral inhibition and inhibitory control are two separate constructs that are both relevant to understanding psychiatric disorders, but their relationship is poorly understood. Behavioral inhibition is a temperament typically manifesting in childhood in which individuals are slow to approach novel objects or unfamiliar people and may interact with inhibitory control to predict the development of disorders such as social anxiety disorder [33], but there is little research investigating the relationship between behavioral inhibition and subcomponents of inhibitory control. We found that BIS score is negatively associated with residualized *K*_d_, suggesting that *excessive* behavioral inhibition is related to a *deficit* in one subcomponent of inhibitory control (involving adjusting for anticipated error). This result could help clarify previous contradictory findings regarding the relationship between anxiety and inhibition. By contrast, the color-word inhibition score was positively related to *K*_p_, suggesting that individuals with greater reactive inhibitory capacity were able to exhibit greater drive on the motor control task. Taken together, our results suggest that parsing inhibition into separate components related to the processing of current error vs. anticipated future error may clarify patterns of inhibition in psychiatric disorders.

Recognition of the involvement of both *K*_p_ and *K*_d_ (drive and damping) enables a more nuanced, process-oriented view of the nature of inhibition in real-time control. While fully separable conceptually and in simulations, the two parameters are positively correlated empirically because higher levels of drive require higher levels of damping to prevent overshoot past the goal state [16]. Drive involves adjusting acceleration on the basis of the current position, while damping involves adjusting acceleration on the basis of current velocity. Because velocity changes more quickly than position, damping may present a greater challenge for the nervous system and represent a more fundamental capacity limitation (which may be influenced by both trait- and state-related factors) that determines what level of drive is possible without overshoot. Importantly, we previously found that damping, controlling for a drive, was negatively associated with self-reported fear [16]. Our present findings demonstrate a similar pattern in which damping, controlling for a drive, was positively associated with neural activations in the stop signal task. This suggests that damping may be a more fundamental process underlying inhibitory control deficits related to psychiatric disorders. Future research can continue to disentangle the roles of these two related components of real-time inhibitory control.

The present finding of the relationship between damping and response inhibition in a transdiagnostic sample suggests that the damping parameter could be useful for representing the capacity of inhibitory processing in clinical populations who tend to show inhibitory deficits, such as substance use disorders, ADHD, OCD, and others. The possibility of measuring core mechanistic processing dysfunctions across diagnoses is consistent with the National Institute of Mental Health (NIMH) Research Domain Criteria (RDoC) framework aiming to develop a dimensional psychiatric classification system [34].

The PD control model is an extension of computational psychiatry, which is often focused on learning and decision-making, to motor control. Motor control requires rapid, real-time decisions that balance competing objectives while accounting for uncertainty [15]. Furthermore, the neural systems underlying motivational and affective influences on decision-making play a similar role in motor control. For example, serotonin and striatal dopamine modulate movement vigor in accordance with reward and effort expectations [35], while the insula [36] and dACC [17] also mediate the planning of movements. NIMH has recognized the importance of sensorimotor processes in understanding psychiatric disorders by adding a sensorimotor RDoC domain [37]. Our results suggest that real-time sensorimotor tasks can serve as data-rich and ecologically valid paradigms for reliable computational assessments of core dysfunctions in inhibitory control across psychiatric disorders.

In this study, our focus was on individual measurement of inhibitory control capacity in individuals with a range of mental health complaints as well as healthy individuals. Given the potential role of inhibitory control deficits across multiple diagnostic categories, as well as variance in the general population, our approach is in line with the dimensional, transdiagnostic RDoC framework [34]. Our research design was not well-powered to detect differences between different diagnostic groups, which would represent a statistical interaction given our focus on the relationship between PD model parameters and neural activations. However, the clinical relevance of the PD model parameters is supported by our previous finding that PD model parameters are related to self-reported fear [16]. Future work can build on the present validation of the PD control framework by applying it to important clinical questions. In particular, future studies can use the PD control framework to examine the effects of interventions designed to improve inhibitory control capacity, thus contributing to improved assessments of treatments for a range of psychiatric disorders.

Limitations of the present study include that the motor task was not performed in an fMRI scanner, although the same participants performed the stop signal task in the scanner. Future studies will use an fMRI version of the motor task to examine the functional neural underpinnings of motor drive and damping more directly. A second limitation is a cross-sectional design. Longitudinal studies can more clearly disentangle trait and state variability in damping capacity and determine whether the PD model can serve as a reliable marker of cognitive control within individuals. Finally, future work can apply more complex paradigms and modeling approaches to more fully explore the connections between motor control, reinforcement learning, and Bayesian decision theory in psychiatric populations.

In conclusion, we found behavioral and imaging evidence for a relationship between motor control (measured with a PD control model) and discrete inhibitory control on a stop signal task. The results suggest that motor control performance, and particularly damping capacity, can serve as a marker for inhibitory control and for the functioning of a number of brain regions involved in cognitive control including the fronto-parietal network. The statistically reliable nature of the PD model parameters suggests that this approach may be pragmatically useful for individualized clinical assessments. Computational motor control paradigms should be further explored as a promising avenue to investigate core domains of dysfunction across a range of psychiatric disorders.

## Supplementary information

Supplemental Material
