# Supplementary material for: Neural substrates of continuous and discrete inhibitory control
Source: Transl Psychiatry. 2023 Jan 24;13:23. doi: 10.1038/s41398-022-02295-0 (PMC9873791; doi:10.1038/s41398-022-02295-0)
Supplement: Supplementary file 1 — Supplemental Material [file 41398_2022_2295_MOESM1_ESM.docx]

**Supplemental Methods**

**Reliability of Parameter Estimation**

We conducted a split-half reliability analysis by separately computing mean *K*_p_ and *K*_d_ values for odd and even trials for each subject. We additionally computed residual *K_d_*, controlling for *K_p_* (in a linear regression model), separately for odd and even trials for each subject. We calculated reliability by computing Pearson correlations between odd-trial and even-trial estimates of *K_p_*, *K_d_*, and residual *K_d_* controlling for *K_p_*.

**Simulations and Parameter Recovery**

In order to test the ability of the model to estimate the parameters, we performed simulations and parameter recovery. We generated a set of 100 simulated trials with varying *K*_p_ and *K*_d_ (randomly permuted such that *K*_p_ and *K*_d_ were not correlated, to test the ability of the model to recover these parameters independently). Within the simulated trials, acceleration at each time point was generated randomly according to a normal distribution with mean determined by the PD control equation and standard deviation based on the estimated standard deviation of residuals from the linear regression model fit to empirical data from subjects in the sample. We then estimated *K*_p_ and *K*_d_ for each simulated trial using the PD model implemented in R.

**Supplemental Results**

**Reliability of Parameter Estimation**

The correlation between the estimate of *K*_p_ based on odd and even trials was r = .98 (Supplemental Figure 3a). The correlation between the estimate of *K*_d_ based on odd and even trials was r = .95 (Supplemental Figure 3b). The correlation between the residual *K*_d_ estimate (controlling for *K*_p_ estimate) based on odd and even trials was r = .89 (Supplemental Figure 3c).

**Simulations and Parameter Recovery**

For simulations, the correlation between true *K*_p_ and recovered *K*_p_ was r = .99 (Supplemental Figure 4a). The correlation between true *K*_d_ and recovered *K*_d_ was r = .97 (Supplemental Figure 4b).

Supplemental Table 1. Brain regions showing the relationship between *K_d_* and Hard control

|  | Peaks (x,y,z) | | | # of voxels | Region | *t*-statistic |
| --- | --- | --- | --- | --- | --- | --- |
| SSSD vs, Go | 3 | 23 | 47 | 237 | R ventromedial frontal cortex | 5.30 |
|  | -33 | 21 | -11 | 112 | L anterior insula | 4.82 |
|  | -53 | -45 | 49 | 159 | L inferior parietal gyurs | 4.84 |
|  | 13 | 17 | 5 | 156 | R caudate | 4.88 |
|  | 57 | -45 | 47 | 142 | R inferior parietal gyrus | 5.21 |
|  | 55 | 11 | 17 | 133 | R medial frontal gyrus | 4.59 |
|  | 41 | 21 | -9 | 128 | R anterior Insula | 4.29 |
|  | -47 | 5 | 25 | 106 | L precentral gyrus | -3.76 |
|  | -39 | -57 | 57 | 84 | R superior parietal lobule | 4.29 |
|  | -3 | -91 | 35 | 81 | L calcarine | 4.62 |
|  | 41 | 3 | 37 | 65 | R medial frontal gyrus | 4.25 |

Supplemental Figure 1


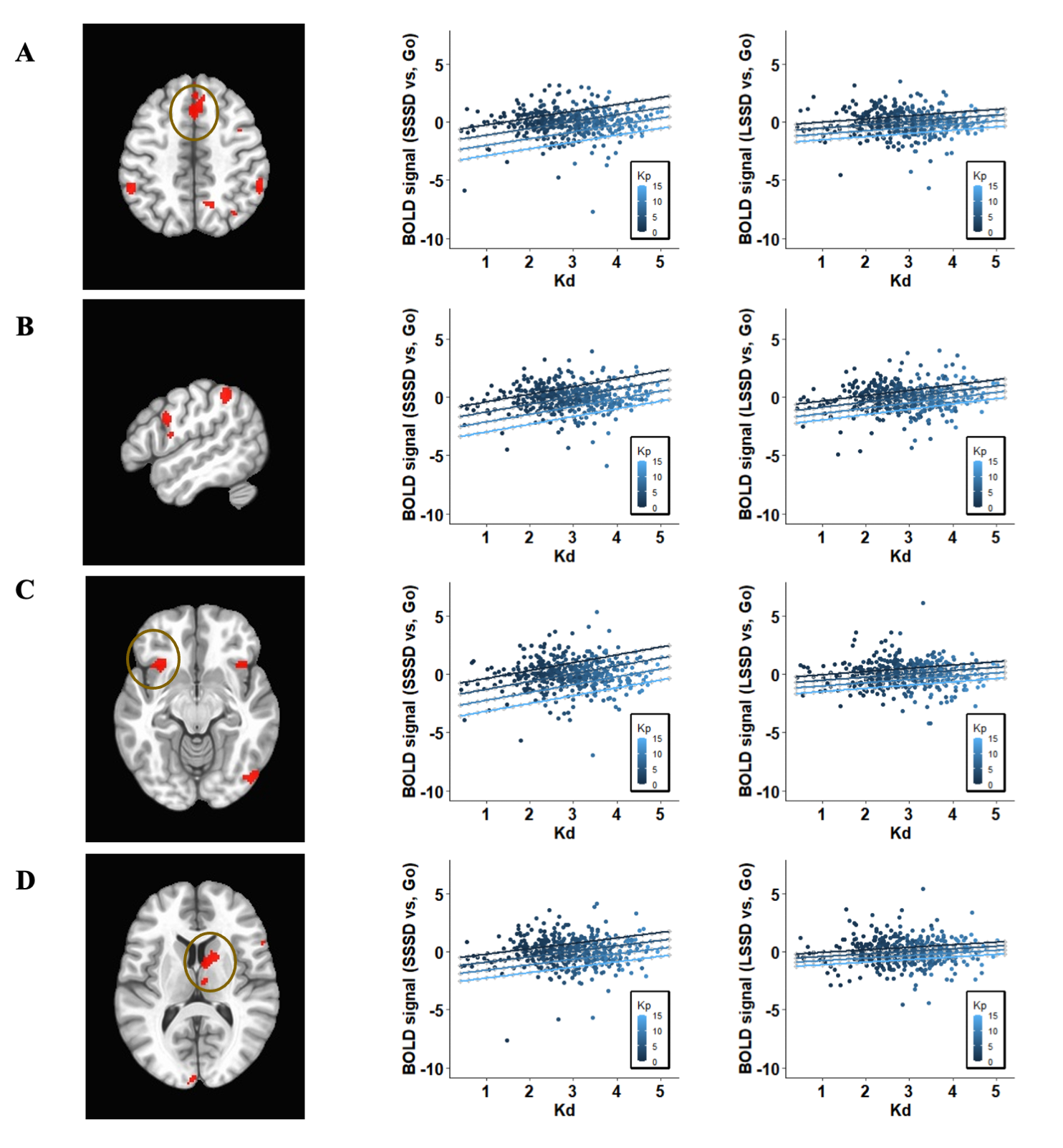


Supplemental Figure 1: Difficult (SSSD > Go) and easy (LSSD > Go) control in the Stop > Go clusters. A: R inferior frontal gyrus; B: L Inferior parietal sulcus C: L aInsula D: R Caudate. SSSD: short stop signal delay or difficult control; LSSD: long stop signal delay or easy control.

Supplemental Figure 2


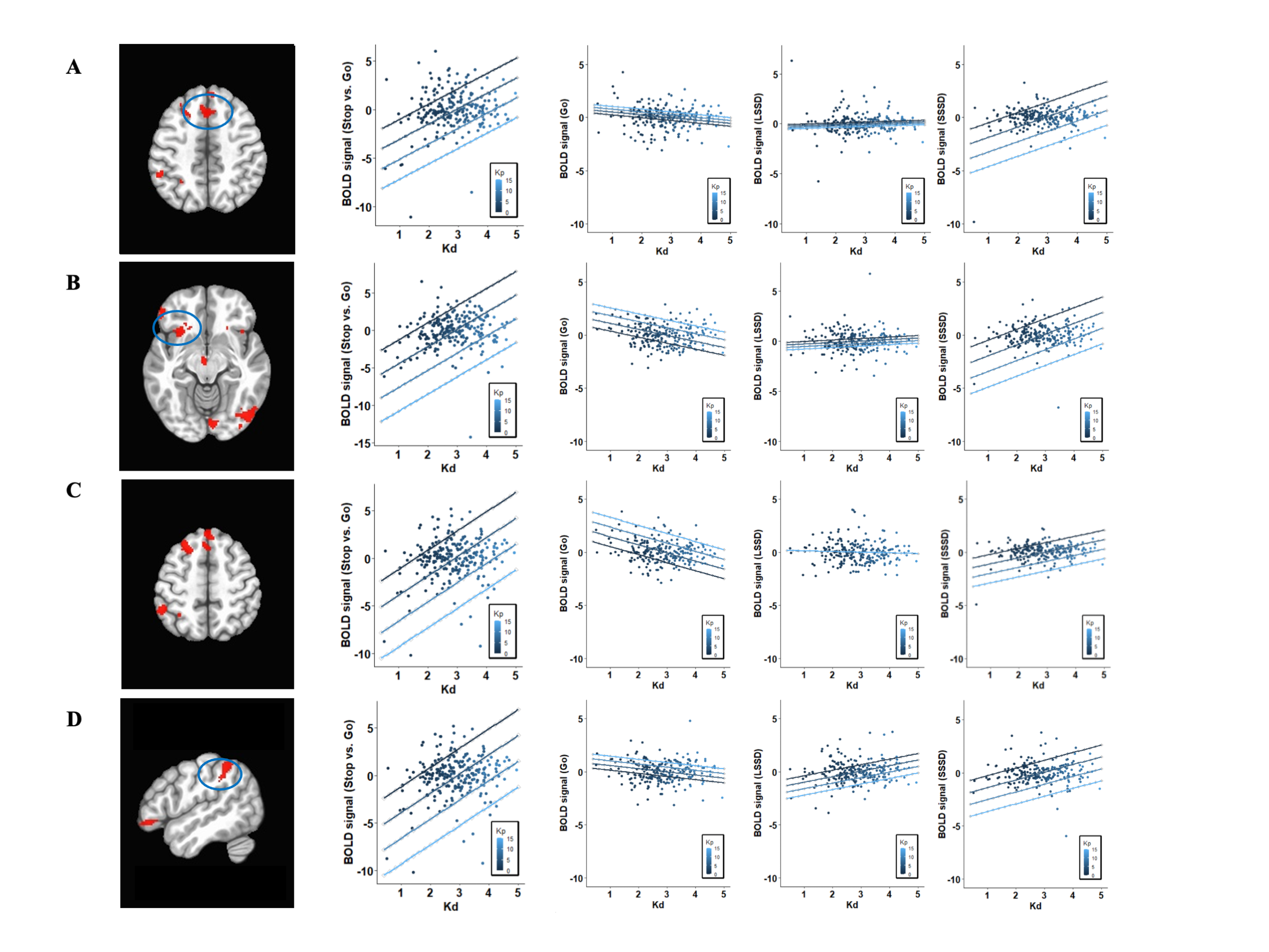


Supplemental Figure 2: Brain regions showing associations between *K_d_* and response control (Stop vs. Go) in individuals with mood disorders. A: Superior medial frontal, B: L anterior insula, C: L middle frontal, D: L inferior parietal lobe. SSSD: short stop signal delay or difficult control; LSSD: long stop signal delay or easy control.

Supplemental Figure 3


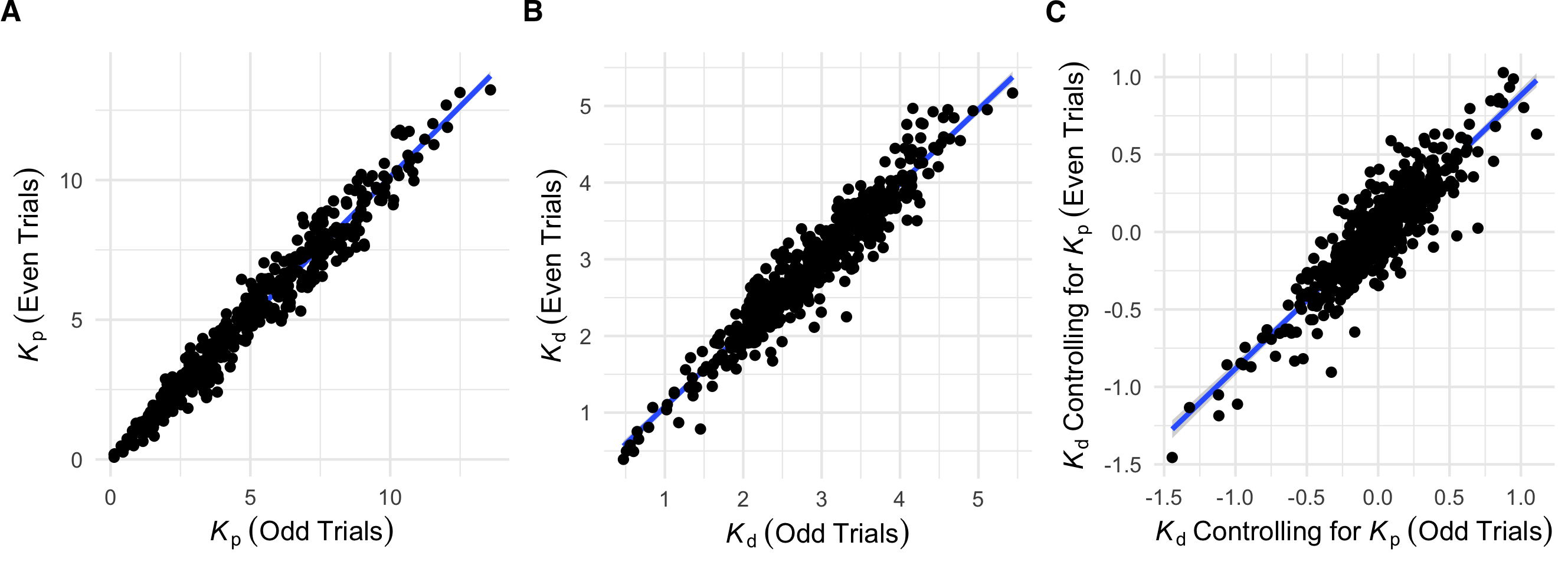


Supplemental Figure 3: Reliability of parameter estimates. A: Split-half reliability of *K*_p_ estimates (odd vs. even trials). B: Split-half reliability of *K*_d_ estimates (odd vs. even trials). C: Split-half reliability of residual *K*_d_ after controlling for *K*_p_ (odd vs. even trials).

Supplemental Figure 4


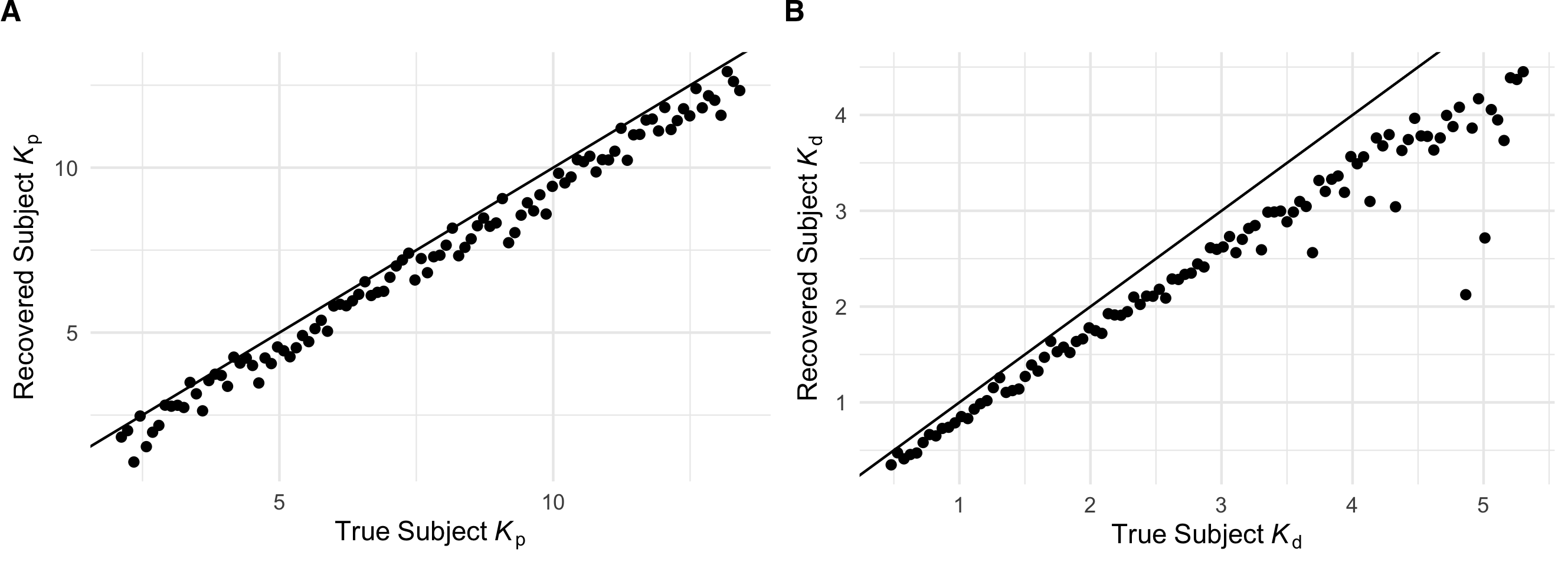


Supplementary Figure 5: Simulations and parameter recovery. A: Recovery of *K*_p_. The diagonal black line represents perfect parameter recovery, i.e. recovered value equal to the true value. B: Recovery of *K*_d_. *K*_p_ and *K*_d_ values were permuted (i.e. were not correlated) to determine whether these parameters could be recovered independently of each other.
